# Irreversible work and Maxwell demon in terms of quantum thermodynamic force

**DOI:** 10.1038/s41598-021-81737-z

**Published:** 2021-01-27

**Authors:** B. Ahmadi, S. Salimi, A. S. Khorashad

**Affiliations:** 1 Department of Physics, University of Kurdistan, P.O.Box 66177-15175, Sanandaj, Iran; 2 International Centre for Theory of Quantum Technologies, University of Gdansk, Wita Stwosza 63, 80-308 Gdansk, Poland

**Keywords:** Physics, Quantum physics, Quantum information

## Abstract

The second law of classical equilibrium thermodynamics, based on the positivity of entropy production, asserts that any process occurs only in a direction that some information may be lost (flow out of the system) due to the irreversibility inside the system. However, any thermodynamic system can exhibit fluctuations in which negative entropy production may be observed. In particular, in stochastic quantum processes due to quantum correlations and also memory effects we may see the reversal energy flow (heat flow from the cold system to the hot system) and the backflow of information into the system that leads to the negativity of the entropy production which is an apparent violation of the Second Law. In order to resolve this apparent violation, we will try to properly extend the Second Law to quantum processes by incorporating information explicitly into the Second Law. We will also provide a thermodynamic operational meaning for the flow and backflow of information. Finally, it is shown that negative and positive entropy production can be described by a quantum thermodynamic force.

## Introduction

Thermodynamics and information have intricate inter-relations. Soon after establishing the second law of thermodynamics by Rodulf Clausius, Lord Kelvin and Max Planck^1–4^, in his 1867 thought experiment, ”Maxwell’s Demon”, James Clerk Maxwell attempted to show that thermodynamics is not strictly reducible to mechanics^5–7^. Although Maxwell introduced his demon to question the Second Law his demon, in fact, revealed that information can be used to perform more work than what is expected by the Second Law. Maxwell illustrated that by using information about the positions and momenta of the particles restrictions imposed by the Second Law can be relaxed thus demanding to take into account information in the Second Law explicitly. In order to do this we must elucidate the physical nature of information so that the Second Law includes information as a physical entity. In 1929 Léo Szilárd^8^, inspired by Maxwell’s idea, designed an engine working in a cycle, interacting with a single thermal reservoir, which used information (gained by the measurement on the system) to perform work.

In equilibrium classical thermodynamics the Clausius’ statement of the Second Law implies that heat only flows from a hot object to a cold one^1–4^. This is actually equivalent to the fact that in an irreversible deterministic process the entropy production of a system is always positive which means that due to irreversibility inside the system information always flows out of the system. This can be readily proved as follows. Clausius showed that the total change in the entropy of a thermodynamic system can be written in the form^1–4^

1 $$\begin{aligned} dS=\dfrac{dQ}{T}+d_iS, \end{aligned}$$

where *dQ* is the heat that the system exchanges with the environment and *T* the temperature of the system and $d_iS$ the entropy produced in the interior of the system which is referred to as entropy production. Now consider two classical thermodynamic systems *A* and *B* which are in equilibrium states with temperatures $T_A$ and $T_B$, respectively. The two systems start exchanging heat quasi-statically, i.e., they remain in equilibrium state while exchanging heat. Assume that the whole system *AB* is closed therefore $dQ_{AB}=0$. Thus using Eq. (1) the change in the entropy of the total system *AB* reads

2 $$\begin{aligned} dS_{AB}=\dfrac{dQ_{AB}}{T_{AB}}+d_iS_{AB}=d_iS_{AB}. \end{aligned}$$

Since entropy is an extensive property we have^1–4^

3 $$\begin{aligned} dS_{AB}=dS_{A}+dS_{B}=\dfrac{dQ_{A}}{T_A}+\dfrac{dQ_{B}}{T_B}, \end{aligned}$$

where $dQ_{A(B)}$ is the heat that system *A*(*B*) exchanges with system *B*(*A*). Note that temperatures of both systems are not constant over time but since the process happens quasi-statically the temperatures of both systems are well-defined at any time. Now since the whole system *AB* is closed then $dQ_{A}=-dQ_{B}$ and if $T_A<T_B$ Eq. (3) becomes

4 $$\begin{aligned} dS_{AB}=dQ_{A}(\dfrac{1}{T_A}-\dfrac{1}{T_B})\ge 0. \end{aligned}$$

Therefore using Eq. (2) for a closed system we get

5 $$\begin{aligned} d_iS_{AB}\ge 0. \end{aligned}$$

It is worth mentioning that the Second Law already implicitly contained the role of information. The positivity of entropy production of a system implies that the system always tends to lose information about its internal energy and this loss of information does not let us to extract the maximum work. This is formulated as^1–4^

6 $$\begin{aligned} dF=dU-TdS=dW-Td_iS, \end{aligned}$$

where the change in Helmholtz energy *dF* is the maximum extractable work from the system and *dU* the change in the internal energy of the system and *dW* the extracted work and $d_iS$ the entropy production of the system during the process. The second equality in Eq. (6) is derived using the first law of thermodynamics $dU=dW+dQ$ and $dS=dQ/T+d_iS$. As can be seen from Eq. (6) positive entropy production $d_iS$ (loss of information) does not let the extracted work *dW* be equal to the maximum extractable work *dF*. But in quantum processes the reversal of energy flow may occur, i.e., heat may flow from the cold system to the hot system that is a violation of the Second Law. In Ref.^9^, using initial quantum correlations between two qubits, the reversal of energy flow between two quantum-correlated qubits is investigated. In quantum stochastic processes there also exist processes, called non-Markovian processes, in which information can backflow into the system therefore it is expected that entropy production of the system becomes negative and consequently again a violation of the Second Law may occur for these processes. These results highlight the subtle interplay of quantum mechanics, thermodynamics and information theory. In this work, we aim to resolve this issue by properly extending the Second Law from equilibrium classical thermodynamics into non-equilibrium quantum thermodynamics such that these violations never occur. For this purpose, we will try to incorporate information explicitly into the Second Law. We will also clarify why and how backflow of information can affect the efficiency of a quantum heat engine. Our results also provide a thermodynamic operational meaning for negative entropy production, which until now only had information-theoretical interpretations; for example, it witnesses the non-Markovianity of a process^10^. It will also be shown that a quantum thermodynamic force^12^ is responsible for the flow and backflow of information.

## Classical engine and its limitation

**Figure 1:**
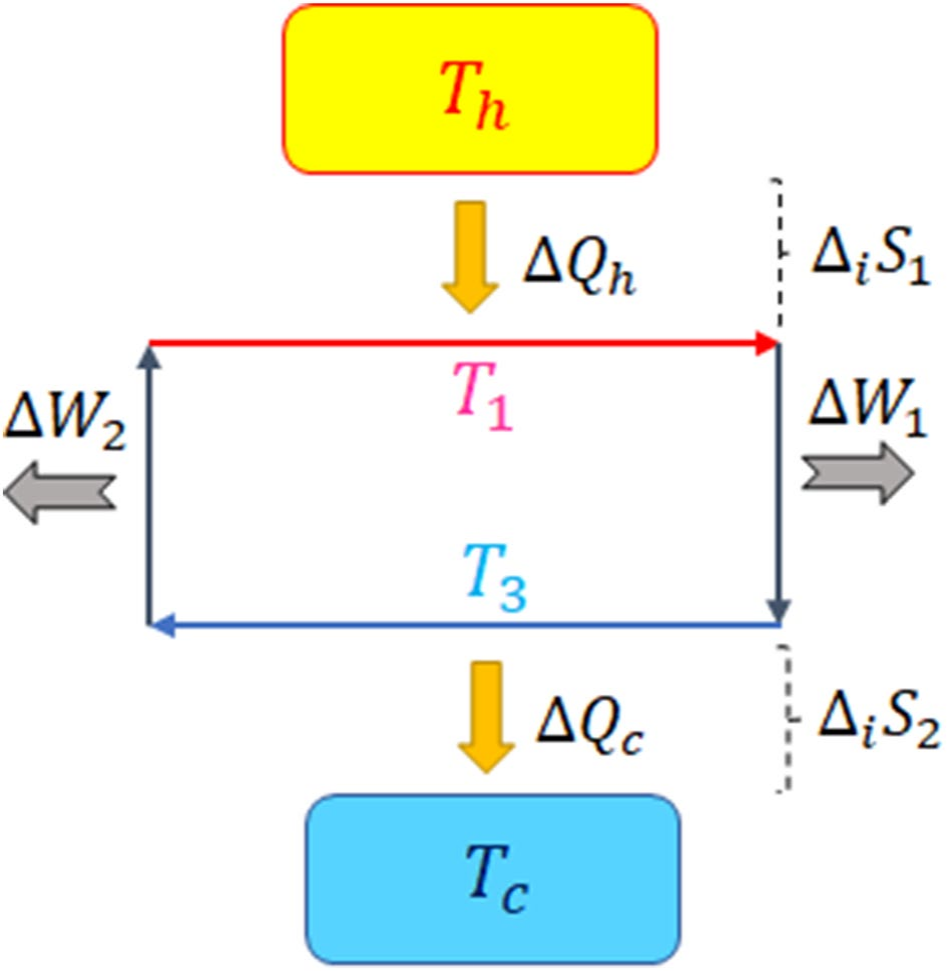
A classical engine working between two reservoirs at temperatures $T_h>T_c$. Irreversibility occurs between the engine and the reservoirs not in the interior of the engine.

In this section we examine a classical engine (see Fig. 1) which gives us the motivation of investigating a quantum heat engine in the presence of backflow of information. For this engine we obtain (see Supplementary Note 1)

7 $$\begin{aligned} \eta _e-\eta _C=-\dfrac{T_c\Delta _iS}{\Delta Q_h}, \end{aligned}$$

where $\eta _e=1-T_3/T_1$ is the engine efficiency, $\eta _C=1-T_c/T_h$ the Carnot efficiency and $\Delta _iS=\Delta _iS_1+\Delta _iS_2$ the entropy production of the total system (the engine plus the reservoirs) during a cycle. Based on the second law of classical thermodynamics the entropy production is always positive thus $\eta _e$ can never exceed $\eta _C$. This is, in fact, equivalent to the relation $T_c\le T_3\le T_1\le T_h$ which always holds for classical engines. Now the question is: does this relation also always hold for quantum heat engines? In the following sections we will address this question.

## Reversible and Irreversible work

The work done by a thermodynamic system, in the weak coupling limit, can always be appropriately partitioned into two parts: reversible work and irreversible work (see Supplementary Note 2),

8 $$\begin{aligned} \Delta W=\Delta W_{rev}+\Delta W_{irr}, \end{aligned}$$

in which

9 $$\begin{aligned} \Delta W_{rev}\equiv \dfrac{1}{\beta }\Delta I+\Delta F^\beta ,\ \quad I(t)=S(\rho _t\Vert \rho ^\beta _t), \end{aligned}$$

and

10 $$\begin{aligned} \Delta W_{irr}\equiv \dfrac{1}{\beta }\Delta _iS. \end{aligned}$$

It is seen that the total work can always be partitioned into two parts, the reversible part and the irreversible part. The reversible work defined in Eq. (9) is different from the definition of reversible work in the literature by the term $\dfrac{1}{\beta }\Delta I$. Quantum relative entropy $I(t)=S(\rho _t\Vert \rho ^\beta _t)$ is a measure of distinguishability between two quantum states^11^, here between the state of the system $\rho _t$ at time *t* and its instantaneous Gibbs state $\rho ^\beta _t$. Quantum relative entropy is in fact the quantum mechanical analog of Kullback–Leibler divergence^11^. In other words, it is a measure of information about the state of the system relative to its instantaneous Gibbs state. Classical thermodynamics is the equilibrium thermodynamics and the minimal work can be extracted only in equilibrium processes, hence the minimal work equals the equilibrium work, i.e., $\Delta W_{min}=\Delta F^\beta$. We must note that $\Delta W_{rev}$ and $\Delta W_{irr}$ in Eq. (8) are not done by the system during the process. The work which is done by the system is $\Delta W$. $|\Delta W_{rev}|$ is the maximal amount of internal energy which is supposed to be spent by the system as work if there was no irreversibility during the process and positive $\Delta W_{irr}$ is the amount of internal energy which is not allowed to be spent by the system as work due to irreversibility (loss of information) inside the system. From now on we will refer to positive $\Delta W_{irr}$ as the *encoded* internal energy because the system does not access this amount of energy to use it as work. This encoded internal energy is always directly related to entropy production via Eq. (10). In other words, we can say

11 $$\begin{aligned} \Delta _iS=\beta \times (encoded\ internal\ enery). \end{aligned}$$

We can go further and define a non-equilibrium free energy for a generic statistical state $\rho$ of a quantum system in contact with a thermal bath as

12 $$\begin{aligned} F(\rho , H)\equiv E-TS=tr\{\rho H\}-TS(\rho ), \end{aligned}$$

and we find

13 $$\begin{aligned} \dfrac{1}{\beta }\Delta _iS=\Delta W_{irr}=\Delta W-\Delta F. \end{aligned}$$

As can be seen from Eq. (13) the minimal work necessary to drive the system from one arbitrary state to another is the difference between the non-equilibrium free energies $\Delta F$. If the entropy production is positive, $\Delta _iS\ge 0$, then the generalized minimal work formulation (the generalized second law) for an isothermal process with given initial and final non-equilibrium distributions is obtained as

14 $$\begin{aligned} \Delta W\ge \Delta F^\beta +\dfrac{1}{\beta }\Delta I. \end{aligned}$$

The generalized minimal work formulation of thermodynamics for non-equilibrium distributions gives an important relation between two major concepts in physics, energy and information. In the following we will see that in non-equilibrium quantum thermodynamics the internal energy can also be decoded (negative irreversible work) to be used by the system to perform more work than what is expected.

## Irreversible work and the Second Law

Let us consider a system in state $\rho _0$ at time $t=0$ attached to a bath of temperature *T*. After a finite-time $\tau$, let the state of the system be $\rho _\tau$. The Hamiltonian *H* of the system remains unchanged during the evolution. The irreversible work after time $\tau$ reads (see Supplementary Note 3)

15 $$\begin{aligned} \Delta W_{irr}=\dfrac{1}{\beta }[S(\rho _0\Vert \rho ^\beta )-S(\rho _\tau \Vert \rho ^\beta )]. \end{aligned}$$

During a Markovian evolution $\Delta W_{irr}$ is always positive but for a non-Markovian evolution it can be negative and this may lead to results not encountered in classical thermodynamics. In the following we focus our attention on two special cases to elucidate the physical meaning of the relation $\Delta W_{irr}=\dfrac{1}{\beta }\Delta _iS$ in non-equilibrium quantum thermodynamics:

(a) Consider a reversible cycle with a quantum engine operating between two heat reservoirs at temperatures $T_h$ and $T_c$ with $T_h>T_c$. Since all the processes are reversible then the work done by the system is $\Delta W=\Delta W_{rev}$. For a machine to work as an engine we should have $\Delta W_{rev}<0$ and since the cycle is reversible, $\Delta W_{rev}= T_h\Delta S_h+T_c\Delta S_c$, the efficiency of the engine equals the Carnot efficiency,

16 $$\begin{aligned} \eta \equiv \dfrac{-\Delta W}{\Delta Q_h}= 1-\dfrac{T_c}{T_h}=\eta _C, \end{aligned}$$

where $\Delta Q_h$ is the heat absorbed from the hot reservoir. Eq. (16) holds for all reversible cycles with classical or quantum heat engines^13^. In equilibrium thermodynamics the Clausius’ statement of the Second Law leads to the fact that of all the heat engines working between two given temperatures, none is more efficient than a Carnot engine^1–3^. As can be seen from Eq. (9) the reason behind this is that in equilibrium thermodynamics, due to the Clausius’ statement of the Second Law, the entropy production $\Delta _iS$ can never be negative, thus it can never help $-\Delta W$ to increase, i.e., the production of entropy is an indication of a reduction in the thermal efficiency of the engine. The Clausius’ statement of the Second Law means that information can never be decoded in deterministic thermodynamics. As we will show below, in stochastic quantum thermodynamics, this is also true as long as the process is Markovian. But if the process is non-Markovian the rate of entropy production can be negative $\dot{S_i}\le 0$^10,14^ consequently we may have negative entropy production for such processes, i.e.,

17 $$\begin{aligned} \Delta _iS=\int _{0}^{\tau }dt\ \dot{S_i}\le 0. \end{aligned}$$

**Figure 2:**
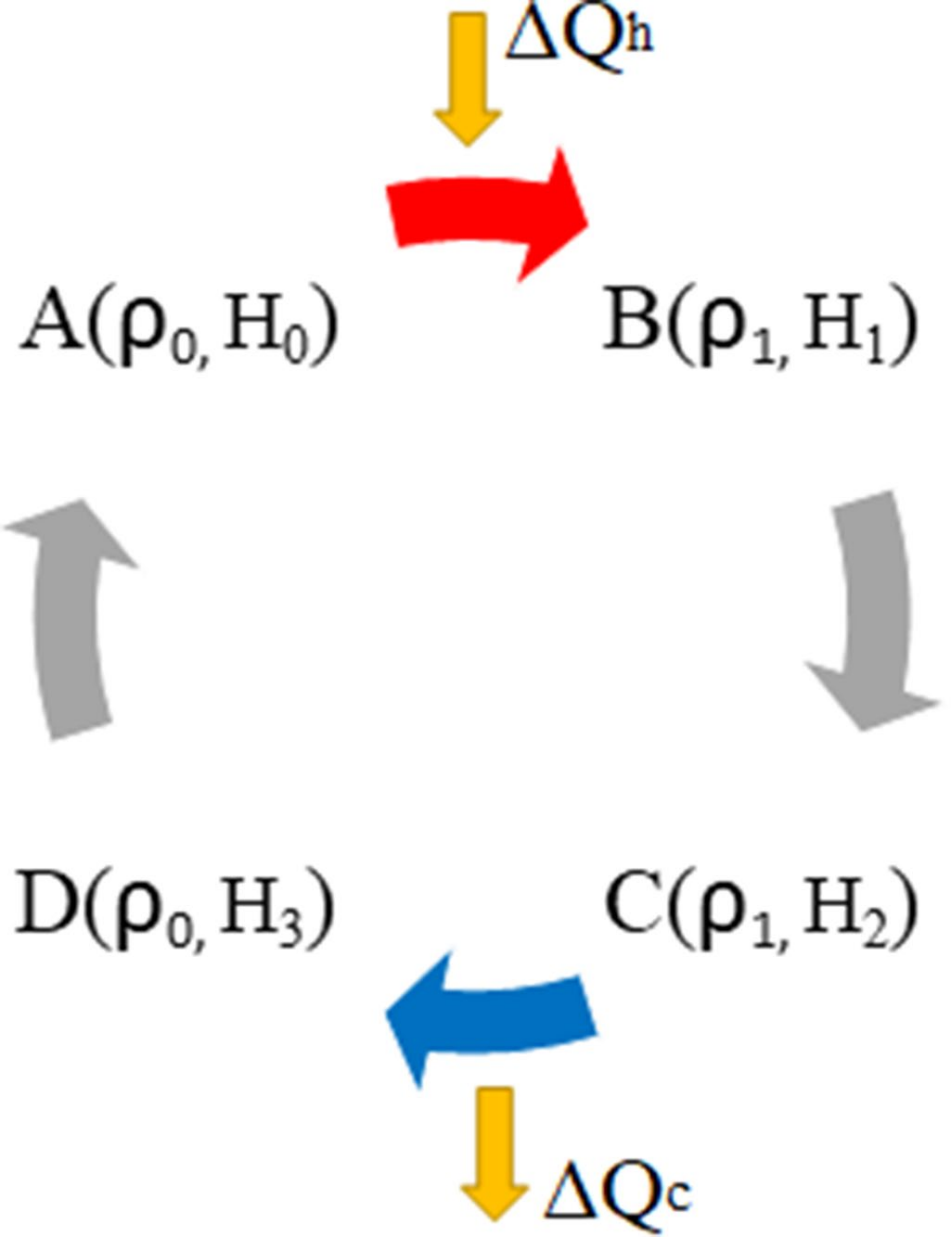
As a visual aid points, between which the quantum system operates as the working substance in an Otto cycle, in dynamical configuration space are depicted in a $(\rho , H)$-coordinate system. From *A* to *B* the heat $\Delta Q_h$ is absorbed from the hot reservoir at temperature $T_h$ and from *C* to *D* the heat $\Delta Q_c$ is rejected to the cold reservoir at temperature $T_c$. The processes from *B* to *C* and from *D* to *A* occur adiabatically.

This means that some of the internal energy can be decoded to be used by the system to do more work. (b) Consider a quantum engine operating in a cycle between two heat reservoirs at temperatures $T_h$ and $T_c$ with $T_h>T_c$. In step I, as depicted in Fig. 2, the engine interacts with a hot reservoir at temperature $T_h$ from point $A(\rho _0, H_0)$ to point $B(\rho _1, H_0)$ while the Hamiltonian remains unchanged. The heat absorbed by the engine is $\Delta Q_h=tr\{H_0(\rho _1-\rho _0)\}$. In step II the engine is decoupled from the hot reservoir and undergoes an adiabatic evolution from point $B(\rho _1, H_0)$ to point $C(\rho _1, H_1)$. In step III it interacts with a cold reservoir at temperature $T_c$ from point $C(\rho _1, H_1)$ to point $D(\rho _0, H_1)$ while the Hamiltonian remains unchanged. The heat rejected to the cold reservoir is $\Delta Q_c=tr\{H_1(\rho _0-\rho _1)\}$. Finally in step IV the engine is decoupled from the cold reservoir and, in an adiabatic evolution, goes back to its initial point by going from point $D(\rho _0, H_1)$ to point $A(\rho _0, H_0)$ and complete the cycle. Now the whole work done by the system during the cycle, as in Eq. (9), is $\Delta W=\Delta W_{irr}+\Delta W_{rev}$. Since during the adiabatic processes no entropy is produced in the interior of the system^13,15^ thus

18 $$\begin{aligned} \Delta W_{irr}=\dfrac{1}{\beta _h}\Delta _iS_h+\dfrac{1}{\beta _c}\Delta _iS_c. \end{aligned}$$

Then the efficiency becomes

19 $$\begin{aligned} \eta = \dfrac{-\Delta W_{rev}}{\Delta Q_h}-\dfrac{\dfrac{\Delta _iS_h}{\beta _h}+\dfrac{\Delta _iS_c}{\beta _c}}{\Delta Q_h}. \end{aligned}$$

Equation (19) is a generic form for the efficiency of any engine working in a cycle. In the case of a reversible cycle the second term on the right hand side vanishes and it simply reduces to Eq. (16). Now as is clear from Eq. (19) the second term on the right hand side shows the contributions of the Markovianity and non-Markovianity of the processes to the efficiency. If the evolution of the system during steps I and III is Markovian then $\Delta _iS_h$ and $\Delta _iS_c$ are positive and consequently the efficiency decreases which would be less than Carnot efficiency. This means that some information is encoded hence the system cannot use this encoded energy as work during the evolution. But if the evolution of the system during steps I and III is non-Markovian then $\Delta _iS_h$ and $\Delta _iS_c$ can be negative and consequently the efficiency increases which can become greater than that of Carnot. This means that information is decoded and the system uses this decoded information to perform additional work and as a result the efficiency increases. Thus, as was mentioned before, one way to exceed the Carnot efficiency is to have non-Markovian processes during the cycle. As an example, consider a spin-1/2 system^13,16–18^ working in an Otto cycle, as depicted in Fig. 2. The system is in an initial state $\rho _0$, diagonal in the eigenbasis of the Hamiltonian $H_0=(\omega _0/2)\sigma _z$, where $\omega _0=\kappa B$ and $\sigma _z$ is the Pauli matrix. Here $\kappa$ is a constant and *B* is the constant magnetic field applied in the *z* direction on the system. The efficiency of the engine reads (see Supplementary Note 4)

20 $$\begin{aligned} \eta =1-\dfrac{\omega _1}{\omega _0}\le 1-\dfrac{T_3}{T_1}. \end{aligned}$$

For a Markovian process in order to absorb heat from the hot reservoir and reject heat to the cold reservoir we must have $T_c\le T_3\le T_1\le T_h$. Thus $1-T_3/T_1\le 1-T_c/T_h$, i.e., the efficiency is less than Carnot efficiency, and the Second Law is preserved. But in the case of non-Markovian baths, since the effective temperature of the system may not approach the temperature of the bath monotonically therefore during the interaction with the hot bath the temperature of the system can become higher than that of the hot bath and during the interaction with the cold bath its temperature can become lower than that of the cold bath, i.e., we may have $T_3\le T_c\le T_h\le T_1$^18–23^ which can lead to an engine more efficient than that of Carnot, resulting in the violation of the Second Law. This violation, in non-equilibrium quantum thermodynamics, occurs due to the memory effects of the baths which is never observed in classical equilibrium thermodynamics. In classical macroscopic thermodynamics no memory effects are present thus no negative entropy production may occur and this leads to the fact that Carnot efficiency is the maximum efficiency. It should be noted that decoupling the system from the reservoir might cause some energy cost but since we are in the weak coupling limit and turning on and off the interaction occurs very fast compared to the time of the step, this energy cost is negligible, i.e, $E_{int}=tr\{\rho H_{int}\}\simeq 0$. It should also be noted that in the example above we did not use any device to make any measurement to gain information from the state of the system and store this information. We left the system and the bath to themselves.

It must be mentioned that if we use a device to gain and store information from the state of the system then according to the Landauer principle in erasing each bit of information $K_BT\ln 2$ of heat is produced^24^ ($K_B$ is Boltzman constant) which compensates for the negative entropy production saving the Second Law. For instance if *N* bit of information is used by the device we will have

21 $$\begin{aligned} \Delta _iS+\Delta S_{device}=\Delta _iS+NK_BT\ln 2\ge 0, \end{aligned}$$

which is in agreement with the Second Law^25^. In Ref.^26^ a device interacting with two heat reservoirs, a work reservoir, and an information reservoir, which exchanges information but not energy with the device, was investigated. They have found that for cyclic processes in which information is systematically written to the memory, the efficiency can exceed the Carnot limit. In this case the system and the bath are not left to themselves, i.e., the information reservoir acts as Maxwell’s demon which intervenes in the process from the outside to decode information. But for the case of non-Markovian bath, in our model above, nothing intervenes in the process from the outside, i.e., no device is used for gaining and storing information. We leave the system and the non-Markovian bath to themselves and information is decoded just due to the memory effects of the bath.

The two cases considered above help to understand the physical nature of information. In 1991 Rolf Landauer declared that ”information is physical”^24^. Since then, information has come to be seen by many physicists as a fundamental component of the physical world^27–30^. In deterministic equilibrium thermodynamics we could also have negative entropy production. Szilárd showed that information can be used to do work if one permits an intelligent being (demon) to intervene in the process of a thermodynamic system^8^. What Maxwell’s demon does is decoding information and the system uses this decoded information to output more work. Decoding information causes the entropy production of the system to be negative, therefore as we have shown above this causes the system to perform more work and, in turn, this leads to having an efficiency greater than that of Carnot. Del Rio *et. al*^31^ have shown that erasing a system, which is coupled strongly with another system (a quantum memory), may cause the conditional entropy of the system to be negative and this negative entropy will lead to extracting work from the system, thus cooling the environment. Our results provide a thermodynamic operational meaning for negative entropy production, which until now only had information-theoretical interpretations; for example, it witnesses the non-Markovianity. The significance of a general Szilárd engine is that it conjoins thermodynamics and information theory. It shows the usefulness of information for performing some thermodynamic task. Given the important link between the task of work extraction and information theory, as appears in the examples of Maxwell’s demon^32^, the Szilárd engine^8^, and Landauer’s erasure principle^33^, it is becoming more common to consider the nature of information as physical. In classical thermodynamics if we leave the system to itself (i.e. no demon is allowed to intervene) it is impossible to have negative entropy production thus the Carnot engine becomes the most possible efficient engine. But in the quantum realm, due to the existence of the landmark quantum features, even if the system is left to itself, in non-Markovian processes the entropy production of the system can be negative thus the system, working in a cycle, can be more efficient than a Carnot engine.

## The second law of thermodynamics

We are now in a position to properly extend the second law of thermodynamics to quantum thermodynamic processes:

“In a quantum thermodynamic process information can be encoded and also decoded for the system to do work and this encoded (decoded) work equals temperature *T* times entropy production of the system, i.e.,

$$\begin{aligned} \Delta W_{irr}=\left\{ \begin{array}{cc} {\dfrac{1}{\beta }\Delta _iS\ge 0} &{} {\text{ information } \text{ is } \text{ encoded }} \\ {\dfrac{1}{\beta }\Delta _iS\le 0} &{} {\text{ information } \text{ is } \text{ decoded }} \end{array}\right. \end{aligned}$$

where $\beta =1/T$ is the temperature of the reservoir with which the system interacts.”

As can be seen this definition of the Second Law emphasizes on the connection between thermodynamics (work as a thermodynamic variable) and information not on a specific direction for the arrow of time because unlike deterministic classical thermodynamics in stochastic quantum thermodynamics the entropy production can be both positive and negative. This way of defining the Second Law covers both classical and quantum thermodynamics and also incorporates information explicitly into the Second Law, i.e., it is never violated in the quantum realm nor in the presence of a demon intervening in the process. For classical macroscopic deterministic thermodynamics it reduces to the encoded part, i.e., $\Delta _iS\ge 0$. Therefore Carnot’s, Clausius’ and the Kelvin-Planck statements of the Second Law come just as a part of the Second Law, i.e., the encoded part. As we have shown in quantum thermodynamic systems information can be decoded spontaneously without any demon intervening in the process and consequently more work than what is expected can be output. In next section we will show that there is a thermodynamic force which is responsible for decoding and encoding information.

## Maxwell’s demon and quantum thermodynamic force

In Ref.^12^ it was shown that a thermodynamic force is responsible for the flow and backflow of information in quantum processes. For a system, interacting with a bath initially at temperature $\beta =1/T$, the rate of the entropy production can be expressed as^12^

22 $$\begin{aligned} \dfrac{d_iS}{dt}=tr\{F_{th}V_{th}\}, \end{aligned}$$

where $V_{th}\equiv {\dot{\rho }}_t\rho _t^\beta$ is the thermodynamic flow and $F_{th}\equiv \dfrac{1}{\rho ^\beta _t}[\ln \rho ^\beta _t-\ln \rho _t]$ the thermodynamic force. Using Eqs. (10) and (22) we get

23 $$\begin{aligned} \dfrac{dW_{irr}}{dt}=\dfrac{1}{\beta } tr\{F_{th}V_{th}\}. \end{aligned}$$

Since it was shown in Ref.^12^ that the thermodynamic force $F_{th}$ is responsible for the flow (encoding) and backflow (decoding) of information in Markovian and non-Markovian dynamics, respectively, Eq. (23) suggests that, if the system is left to itself, $F_{th}$ actually encodes energy, during the flow, not to be used as work by the system and decodes energy, during the backflow, to be used as work by the system. In classical thermodynamics De Donder found a similar relation for chemical reactions^34^. Let us now consider the case in which the system is not left to itself, i.e., someone or something outside the system (as a demon) intervenes in the process. Szilárd argued that negative work $\Delta W$ can be extracted from an isothermal cycle if Maxwell’s demon plays the role of a feedback controller^35^. When the statistical state of a system changes from $\rho (x)$ to $\rho (x|m)$, due to the measurements made by the demon on the system, the change in the entropy of the system can be expressed as^36,37^

24 $$\begin{aligned} \Delta S_{meas}=H(X|M)-H(X)=-I(X:M), \end{aligned}$$

where $H(X)=-\sum _x\rho (x)\ln \rho (x)$ is the Shanon entropy of the system and *I*(*X*; *M*) the mutual information between the state of the system and the measurement outcome *M*. Since *I*(*X*; *M*) is always positive thus the demon causes the entropy of the system to decrease. This is similar to the case of non-Markovianity in which the entropy decreases. Therefore the presence of the demon is also expected to lead to extracting more work from the system than what is expected. Now the role of the demon can be incorporated into the Second Law as^36,38^

25 $$\begin{aligned} \Delta W\ge \Delta F-\dfrac{1}{\beta }I(X:M). \end{aligned}$$

In Ref.^32^ a practical way was offered, as an alternative to the Szilárd engine, to physically realize Maxwell’s demon. They have shown that using a feedback contoller (the demon) which makes measurements on the engine they are capable of extracting more work from the heat reservoirs than is otherwise possible in thermal equilibrium. For a system, initially and finally in equilibrium states with temperature $\beta =1/T$, which can contact heat reservoirs $B_1, B_2, ..., B_n$ at respective temperatures $T_1, T_2, ..., T_n$ they have found that

26 $$\begin{aligned} \Delta W \ge \Delta F^\beta -\dfrac{1}{\beta }I(\rho _1:X), \end{aligned}$$

and

$$\begin{aligned} I(\rho _1:X)=\dfrac{1}{\beta }[S(\rho _1)-H(\{p_k\})+H(\rho _1:X)], \end{aligned}$$

where $\rho _1$ is the state of the system at some time $t_1$, $S(\rho _1)$ the Von Neumann entropy, $H(\{p_k\})=-\sum _{k}p_k\ln p_k$ the Shannon information content and $H(\rho _1:X)=-\sum _{k}tr\{\sqrt{D_k}\rho _1\sqrt{D_k}\ln \sqrt{D_k}\rho _1\sqrt{D_k}\}$. $\{D_k\}$ are positive operator valued-measure (POVM) defined by $D_k=M^\dagger _kM_k$ and $p_k=tr\{D_k\rho \}$. It is seen that the sum of the last three terms on the right hand side of the inequality (26) is the irreversible work due to the presence of the feedback controller (the demon). Thus if we take the time derivative of these three terms we have

27 $$\begin{aligned} \dfrac{dW^{dem}_{irr}}{dt}= & {} \dfrac{1}{\beta }[tr\{\dot{\rho _1}\ln \rho _1\}+\sum _{k}{\dot{p}}_k\ln p_k \nonumber \\- & {} \sum _{k}tr\{\sqrt{D_k}{\dot{\rho }}_1\sqrt{D_k}\ln \sqrt{D_k}\rho _1\sqrt{D_k}\}]. \end{aligned}$$

Comparing Eq. (27) with Eq. (23) it is observed that there are three quantum thermodynamic forces responsible for the extra work done during the process,

28 $$\begin{aligned} F^1_{th}=\dfrac{\ln \rho _1}{\rho ^\beta _1},\ F^{2(k)}_{th}=\dfrac{\ln p_k}{p^\beta _k},\ F^{3(k)}_{th}=-\dfrac{\ln \sqrt{D_k}\rho _1\sqrt{D_k}}{\rho ^\beta _1}. \end{aligned}$$

Thus we may write

29 $$\begin{aligned} F^{tot}_{th}=F^1_{th}\bigoplus F^2_{th}\bigoplus F^3_{th}. \end{aligned}$$

There are also three thermodynamic flows associated with these three thermodynamic forces above,

30 $$\begin{aligned} V^1_{th}=\dot{\rho _1}\rho ^\beta _1,\ V^{2(k)}_{th}={\dot{p}}_kp^\beta _k,\ V^{3(k)}_{th}=\sqrt{D_k}{\dot{\rho }}_1\sqrt{D_k}\rho ^\beta _1, \end{aligned}$$

and it may be written

31 $$\begin{aligned} V^{tot}_{th}=V^1_{th}\bigoplus V^2_{th}\bigoplus V^3_{th}. \end{aligned}$$

We must notice that Eqs. (29) and (31) should not be taken too literally, i.e., these equations just indicate the fact that there are three thermodynamic forces and flows involved due to the presence of the feedback controller and we cannot add them up like the way we do about typical vectors. We note that $F^{tot}_{th}=0$ if and only if $D_k$ is proportional to the identity operator for all *k*^32^, which means that nothing is intervening in the process, therefore no information is decoded to be used to perform additional work by the system. On the other hand, $F^{tot}_{th}=F^2_{th}$ if and only if $D_k$ is the projection operator satisfying $[\rho _1, D_k]=0$ for all *k*^32^, which means that the measurement on $\rho _1$ is classical, hence $F^{tot}_{th}$ is classical. In Refs.^36,39,40^ similar results have been found. Therefore we have shown that intervention (the demon) from the outside in the process of a system may be represented by a thermodynamic force.

## Summary

In this work we have appropriately divided the work done by a thermodynamic system into two parts: reversible work and irreversible work. This partitioning seems plausible since whenever the process is reversible all the work is reversible and there exists no irreversible work as expected. Using this partitioning we have derived a generic form for the efficiency of an engine operating in an arbitrary cycle. It was shown that negative entropy production, which can occur in non-Markovian processes or by intervening in the process of the system (Maxwell’s demon), means that the internal energy is decoded to be used by the system to perform more work than what is expected and this additional work leads to having quantum engines with efficiencies greater than that of Carnot. We have investigated two special cases to elucidate the physical meaning of $\Delta W_{irr}=\dfrac{1}{\beta }\Delta _iS$ in quantum thermodynamics. We have also shown that the relation $\Delta W_{irr}=\dfrac{1}{\beta }\Delta$ is the link between thermodynamics and information in both classical and quantum thermodynamics. Based on this analysis we have introduced a new definition of the second law of thermodynamics such that it covers both classical and quantum thermodynamics and incorporates information well into the Second Law. At last, we have shown that a quantum thermodynamic force is responsible for encoding and decoding information even when a feedback controller outside the system is involved in the process.

## Supplementary Information

Supplementary Information.
