# Supplementary material for: Irreversible work and Maxwell demon in terms of quantum thermodynamic force
Source: Sci Rep. 2021 Jan 27;11:2301. doi: 10.1038/s41598-021-81737-z (PMC7840741; doi:10.1038/s41598-021-81737-z)
Supplement: Supplementary file 1 — Supplementary Information. [file 41598_2021_81737_MOESM1_ESM.pdf]

Irreversible Work and Maxwell Demon in terms of Quantum Thermodynamic Force

B. Ahmadi[1,2,*], S. Salimi[1], and A. S. Khorashad[1]

[1]Department of Physics, University of Kurdistan, P.O.Box 66177-15175, Sanandaj, Iran
[2]International Centre for Theory of Quantum Technologies, University of Gdansk, Wita Stwosza 63, 80-308 Gdansk, Poland
[*]Correspondence and requests for materials should be addressed to B. Ahmadi. (email: b.ahmadi19@gmail.com)

Supplementary Note 1

The entropy production between the engine and the hot reservoir is

$$\Delta_i S_1 = \frac{\Delta Q_h}{T_1} - \frac{\Delta Q_h}{T_h}, \tag{1}$$

and between the engine and the cold reservoir

$$\Delta_i S_2 = \frac{\Delta Q_c}{T_c} - \frac{\Delta Q_c}{T_3}. \tag{2}$$

The total entropy production during a cycle is $\Delta_i S = \Delta_i S_1 + \Delta_i S_2$. Since no irreversibility occurs in the interior of the engine we have

$$\frac{\Delta Q_h}{T_1} = \frac{\Delta Q_c}{T_3}. \tag{3}$$

Thus we get

$$\Delta_i S = \frac{\Delta Q_c}{T_c} - \frac{\Delta Q_h}{T_h}. \tag{4}$$

The efficiency of the engine reads

$$\eta_e \equiv \frac{-(\Delta W_1 + \Delta W_2)}{\Delta Q_h} = 1 - \frac{\Delta Q_c}{\Delta Q_h} = 1 - \frac{T_3}{T_1}. \tag{5}$$

Now combining Eqs. (4) and (5) we obtain

$$\eta_e - \eta_C = -\frac{T_c \Delta_i S}{\Delta Q_h}, \tag{6}$$

where $\eta_C = 1 - \frac{T_c}{T_h}$ is the Carnot efficiency.

Supplementary Note 2

Irreversibility in physical process is strictly related to the idea of energy dissipation. Irreversible processes encountered by an open thermodynamic system are accompanied with a production of entropy which is fundamentally different from the entropy flow in the form of heat caused by the interaction between the system and its environment. Characterization of irreversibility is one of the cornerstones of non-equilibrium thermodynamics since the theory was born. For an isothermal process the second law of classical deterministic equilibrium thermodynamics may be expressed as

$$\Delta W \geq \Delta F^{\beta}, \tag{7}$$

where ΔW is the amount of work required to change the state of the system between two equilibrium states and ΔF^{β} the difference in the Helmholtz free energy of the system. This, in turn, led to the introduction of the so-called irreversible work for classical systems[S41, S42]

$$\Delta W_{irr} \equiv \Delta W - \Delta F^{\beta} \geq 0. \tag{8}$$

On the other hand for classical thermodynamic systems we have[S1–S3]

$$\Delta F^{\beta} = \Delta W - \frac{1}{\beta}\Delta_i S. \tag{9}$$

Hence defining ΔW_{irr} as in Eq. (8) gives rise to

$$\Delta W_{irr} = \frac{1}{\beta}\Delta_i S, \tag{10}$$

where $\Delta_i S$ is the entropy production of the system during the irreversible process and $\beta = 1/T$ the temperature of the system. It should be emphasised that in classical equilibrium thermodynamics the reversible work equals the change in the free energy, i.e, $\Delta W_{rev} = \Delta F^{\beta}$[S1–S3]. Thus the total work done by the system is partitioned into reversible and irreversible parts, i.e, $\Delta W = \Delta W_{rev} + \Delta W_{irr}$. Thermodynamic reversibility is achieved if and only if no entropy is produced inside the system, i.e, zero entropy production[S1–S3]

$$\Delta_i S = 0. \tag{11}$$

Eq. (10) has a very subtle and interesting meaning. It links thermodynamics with information theory. It says that some of the internal energy, during the irreversible process, is encoded due to the loss of information and consequently the system cannot use this amount of internal energy to do work. For instance in the process of the free expansion of a gas all the internal energy is encoded therefore no internal energy can be used by the gas to perform any work, i.e, $|\Delta W_{rev}| = |\Delta W_{irr}|$. Thus if the system operates in an irreversible cycle its efficiency decreases[S1–S3]. In other words Eq. (10) means that information is physical. Eqs. (8) and (10) have been extended to quantum thermodynamics with the same formulae[S43–S51]. This may be wrong and misleading for two reasons. First, the inequality (7) does not always hold in the quantum realm. Because in stochastic quantum thermodynamics there exist processes, called non-Markovian processes, in which we may have

$$\Delta W \leq \Delta F^{\beta}. \tag{12}$$

This is because in deterministic classical thermodynamics, according to the Clausius' statement of the Second Law, entropy production of a system can never be negative[S1–S3] but in quantum thermodynamics, during non-Markovian processes, entropy production of the system may be negative[S10, S14]. Second in quantum thermodynamics, as we will show in this work, the reversible work ΔW_{rev} done by a system equals $\frac{1}{\beta}\Delta I + \Delta F^{\beta}$ where $I(t) = S(\rho_t \| \rho_t^{\beta})$. If now we apply Eq. (9) to the evolution of a closed quantum system we observe that $\Delta F^{\beta} - \Delta W$ could be nonzero while $\Delta_i S = 0$,

$$\Delta S = \frac{\Delta Q}{T} + \Delta_i S,\ \Delta S = 0,\ \Delta Q = 0 \ \Rightarrow\ \Delta_i S = 0, \tag{13}$$

where $\Delta S = 0$ because the evolution of a closed system is unitary and $\Delta Q = 0$ because a closed system does not interact with the environment. This means that the evolution of a closed quantum system is reversible even if neither the initial nor the final state of the system is equilibrium. the process is reversible. Thus if ΔW_{irr} as defined in Eq. (8) is extended to quantum thermodynamic systems with the same formulae, for a closed quantum system Eq. (10) may not hold. For instance, in the case of the evolution of a closed quantum system initially in equilibrium we have $\Delta W_{irr} = \frac{1}{\beta}S(\rho_t \| \rho_t^{\beta}) \neq 0$ while $\Delta_i S = 0$.

Reversible and irreversible work. Using the definition of average energy $\langle E \rangle = tr\{\rho_t H_t\}$[S15, S52] for the rate of average energy, where ρ_t is the instantaneous state of the system and H_t the instantaneous Hamiltonian of the system at time t, we have

$$\langle \dot{E} \rangle = tr\{\dot{\rho}_t H_t\} + tr\{\rho_t \dot{H}_t\}, \tag{14}$$

where the first term on the RHS of Eq. (14) is defined as the rate of average heat and the second term as the rate of average work[S53]. Therefore average heat and average work (from time $t_0 = 0$ to $t = \tau$) are respectively defined as

$$\Delta Q \equiv \int_0^{\tau} dt\ tr\{\dot{\rho}_t H_t\}, \tag{15}$$

$$\Delta W \equiv \int_0^\tau dt\ tr\{\rho_t \dot{H}_t\}. \tag{16}$$

Now consider an arbitrary quantum system S coupled with a heat reservoir B at temperature $\beta = 1/T$. Eq. (16) becomes

$$\Delta W = -\frac{1}{\beta}\int_0^\tau dt\ tr\{\rho_t \partial_t \ln \rho_t^\beta\} + \Delta F^\beta, \tag{17}$$

where $\rho_t^\beta = \exp(-\beta H_t)/Z_t$ is the instantaneous Gibbs state of the system with Z_t the partition function and $F_t^\beta = -\frac{1}{\beta}\ln Z_t$ the free energy of the system. The total change in the entropy ΔS of the system is divided into two parts[S1–S3]

$$\Delta S = \Delta_i S + \Delta_e S, \tag{18}$$

in which $S = -tr\{\rho \ln \rho\}$ is the Von Neumann entropy of the system, $\Delta_e S \equiv \beta \Delta Q$ the entropy change due to the exchange of energy with the reservoir and $\Delta_i S$ the entropy produced by the irreversible processes in the interior of the system. In contrast to the thermodynamic entropy that can be defined only for thermal equilibrium, the Von Neuwman entropy can be defined for an arbitrary probability distribution. Combining Eqs. (15)–(18), we get

$$\Delta_i S = S(\rho_0 \| \rho_0^\beta) - S(\rho_\tau \| \rho_\tau^\beta) - \int_0^\tau dt\ tr\{\rho_t \partial_t \ln \rho_t^\beta\}, \tag{19}$$

where $S(\rho\|\sigma) \equiv tr\{\rho \ln \rho\} - tr\{\rho \ln \sigma\}$ is the relative entropy of the states ρ and σ. A thermodynamic reversible process is defined as a process that can be reversed without leaving any trace on the surroundings. That is, both the system and the surroundings are returned to their initial states at the end of the reverse process. This definition of reversibility in conventional thermodynamics may be completely characterized by the entropy production. Thermodynamic reversibility is achieved if and only if the entropy production is zero, i.e., $\Delta_i S = 0$[S1–S3]. A stochastic process is thermodynamically reversible, if and only if the final probability distribution can be restored to the initial one, without remaining any effect on the outside world[S38]. As in conventional thermodynamics, reversibility in stochastic processes is completely characterized by the entropy production. Reversibility in stochastic thermodynamics is achieved if and only if the entropy production is zero[S38], i.e.,

$$\Delta_i S = 0. \tag{20}$$

Theorem 1 *The work done by a thermodynamic system, in the weak coupling limit, can always be appropriately partitioned into two parts: reversible work and irreversible work, i.e,*

$$\Delta W = \Delta W_{rev} + \Delta W_{irr}, \tag{21}$$

in which

$$\Delta W_{rev} = \frac{1}{\beta}\Delta I + \Delta F^\beta, \tag{22}$$

and

$$\Delta W_{irr} = \frac{1}{\beta}\Delta_i S. \tag{23}$$

Proof. Since in a reversible process entropy production is zero using Eqs. (17) and (19), after some straightforward calculations, the (reversible) work is obtained as

$$\Delta W_{rev} = \frac{1}{\beta}\Delta I + \Delta F^\beta, \tag{24}$$

where $I(t) = S(\rho_t \| \rho_t^\beta)$. Unlike the reversible processes, during a general process the entropy may be produced inside the system (irreversible processes), i.e., $\Delta_i S \neq 0$[S15, S52]. Therefore we find

$$\Delta W = \frac{1}{\beta}\Delta_i S + \frac{1}{\beta}\Delta I + \Delta F^\beta, \tag{25}$$

where the first term is the irreversible work ΔW_{irr} and the sum of the last two terms on the right hand side is the reversible work ΔW_{rev}. Hence the total work done by a system during a general process can be expressed as

$$\Delta W = \Delta W_{irr} + \Delta W_{rev}. \quad \square \tag{26}$$

The non-equilibrium free energy for a generic statistical state ρ of a quantum system in contact with a thermal bath is defined as

$$F(\rho, H) \equiv E - TS = tr\{\rho H\} - TS(\rho), \tag{27}$$

where H is the Hamilton of the system. Using Eqs. (15) and (16), Theorem 1, and Eq. (27) we obtain

$$\frac{1}{\beta}\Delta_i S = \Delta W_{irr} = \Delta W - \Delta F. \tag{28}$$

Eq. (28) is the extension of Eq. (8) to quantum thermodynamics. The only difference is that ΔF in Eq. (28) is the difference in non-equilibrium free energies and as we mentioned before this is because quantum thermodynamics is a non-equilibrium thermodynamics. The associated non-equilibrium free energy is analogous to its equilibrium counterpart in non-equilibrium processes. When the initial and final states of the system are thermal equilibrium states Eq. (28) becomes equivalent to Eq. (8) in conventional thermodynamics as expected.

Supplementary Note 3

Consider a system in state ρ_0 at time $t = 0$ attached to a bath of temperature T. After a finite-time τ, let the state of the system be ρ_τ. The Hamiltonian H of the system remains unchanged during the evolution. Therefore, using Eq. (19), the entropy production of the system after a time τ is

$$\Delta_i S = S(\rho_0 \| \rho^\beta) - S(\rho_\tau \| \rho^\beta). \tag{29}$$

For a completely positive, trace preserving (CPTP) map Λ_t and any two density matrices ρ_1 and ρ_2, if the dynamics is Markovian for which $\Lambda_t[\rho^\beta] = \rho^\beta$ for all t, we have[S54]

$$S(\rho_2 | \rho_\beta) = S(\Lambda_t[\rho_1] \| \Lambda_t[\rho^\beta]) \leq S(\rho_1 \| \rho^\beta). \tag{30}$$

But if the dynamics is non-Markovian, since $\Lambda_t[\rho^\beta] \neq \rho^\beta$, we can have[S54]

$$S(\rho_2 \| \rho^\beta) \geq S(\rho_1 \| \rho_\beta). \tag{31}$$

The heat exchanged between the system and the bath is obtained as

$$\begin{aligned} \Delta Q &= T\Delta S - T\Delta_i S \\ &= T[S(\rho_\tau) - S(\rho_0)] + T[S(\rho_\tau \| \rho^\beta) - S(\rho_0 \| \rho^\beta)]. \end{aligned} \tag{32}$$

Supplementary Note 4

Here we consider a spin-1/2 system[S13, S16–S18] working in an Otto cycle, as depicted in Fig. (2) of the main text. The system is in an initial state ρ_0, diagonal in the eigenbasis of the Hamiltonian $H_0 = (\omega_0/2)\sigma_z$, where $\omega_0 = \kappa B$ and σ_z is the Pauli matrix. Here κ is a constant and B is the constant magnetic field applied in the z direction on the system. In step I the engine interacts weakly with a hot reservoir at temperature T_h, for time τ_1 from point $A(\rho_0, H_0)$ to point $B(\rho_1, H_0)$. The final state of the system is $\rho_1 = \exp(-H_0/T_1)/tr\{\exp(-H_0/T_1)\}$, which is diagonal in the eigenbasis of H_1. Here $T_1 = -\omega_0/(2\tanh^{-1}\langle\sigma_z\rangle_1)$, where $\langle\sigma_z\rangle = tr\{\rho_1\sigma_z\}$, is the effective temperature of the system after time τ_1. The heat absorbed by the engine is $\Delta Q_h = tr\{H_0(\rho_1 - \rho_0)\}$. In step II the engine is decoupled from the hot reservoir and undergoes an adiabatic evolution from point $B(\rho_1, H_0)$ to point $C(\rho_1, H_1)$ by varying the magnetic field from ω_0 to ω_1 ($\omega_1 < \omega_0$). Since the system performs work, the temperature of the system changes at the end of this process and it becomes $T_2 = T_1\omega_1/\omega_0$. In step III it interacts weakly with a cold reservoir at temperature T_c from point $C(\rho_1, H_1)$ to point $D(\rho_0, H_1)$ for time τ_2 and the state of the system becomes ρ_0 with the effective temperature $T_3 = -\omega_1/(2\tanh^{-1}\langle\sigma_z\rangle_0)$. The heat rejected to the cold reservoir is $\Delta Q_c = tr\{H_1(\rho_0 - \rho_1)\}$. Finally in step IV the engine is decoupled from the cold reservoir and, in an adiabatic evolution, goes back to its initial point by going from point $D(\rho_0, H_1)$ to point $A(\rho_0, H_0)$ and complete the cycle. The temperature of the system at the end of this cycle becomes

$T_0 = T_3\omega_0/\omega_1$. It can be shown that the effective temperatures of the system approach the temperatures of the heat baths asymptotically[S18]. The heat absorbed by the system from the hot reservoir during step I is given by

$$\Delta Q_h = \frac{\omega_0}{2}[\tanh(\frac{\omega_1}{2T_3}) - \tanh(\frac{\omega_0}{2T_1})]. \tag{33}$$

In the same way, the heat rejected to the cold heat during step III is obtained as

$$\Delta Q_c = -\frac{\omega_1}{2}[\tanh(\frac{\omega_1}{2T_3}) - \tanh(\frac{\omega_0}{2T_1})]. \tag{34}$$

Now the total work done by the system after the cycle is $\Delta W = -(\Delta Q_h + \Delta Q_c)$, i.e,

$$\Delta W = \frac{\omega_1 - \omega_0}{2}[\tanh(\frac{\omega_1}{2T_3}) - \tanh(\frac{\omega_0}{2T_1})]. \tag{35}$$

For a machine to work as an engine we must have $\Delta W < 0$, $\Delta Q_h > 0$ and $\Delta Q_c < 0$. This implies that

$$\frac{\omega_1}{T_3} \geq \frac{\omega_0}{T_1}. \tag{36}$$

Hence the efficiency of the engine reads

$$\eta = 1 - \frac{\omega_1}{\omega_0} \leq 1 - \frac{T_3}{T_1}. \tag{37}$$

References

S1. Kondepudi, D. & Prigogine, I. *Modern Thermodynamics* (New York: Wiley 1998).

S2. Blundell, S. J. & Blundell, K. M. *Concepts in Thermal Physics* (Oxford University Press 2009).

S3. Callen, H. B. *Thermodynamics and an Introduction to Thermostatistics 2nd edn* (JohnWiley, 1985).

S4. Le Bellac, M., Mortessagne, F., & Batrouni, G. G. *Equilibrium and non-Equilibrium Thermodynamics* (Cambridge University Press 2004).

S5. Maxwell, J. C. *Theory of Heat* (Appleton, London, 1871).

S6. Leff, H. S. & Rex, A. F. *Maxwell's Demon: Entropy, Information, Computing* (Princeton Univ. Press, 1990).

S7. Maruyama, K., Nori, F. & Vedral, V. Colloquium: The physics of Maxwell's demon and information. Rev. Mod. Phys. **81**, 1-23 (2009).

S8. Szilárd, L. On the decrease of entropy in a thermodynamic system by the intervention of intelligent beings. Zeitschrift für Physik **53**, 840 (1929).

S9. Micadei, K., Peterson, J. P. S., Souza, A. M. Reversing the direction of heat flow using quantum correlations. *et al.* Nat. Commun. **10**, 2456 (2019).

S10. Hong-Bin Chen, Guang-Yin Chen, & Yueh-Nan Chen, Thermodynamic description of non-Markovian information flux of nonequilibrium open quantum systems. Phys. Rev. A **96**, 062114 (2017).

S11. M. A. Nielsen and I. L. Chuang, Quantum Computation and Quantum Information (Cambridge University Press, 2000).

S12. Ahmadi, B., Salimi, S., & Khorashad A. S. & Kheirandish, F. The quantum thermodynamic force responsible for quantum state transformation and the flow and backflow of information. Sci. Rep. **9**, 8746 (2019).

S13. Quan, H. T., Liu, Y.-x., Sun, C. P. and Nori, F., Quantum thermodynamic cycles and quantum heat engines. Phys. Rev. E **76**, 031105 (2007).

S14. Marcantoni, S., Alipour, S., Benatti, F., Floreanini, R., & Rezakhani, A. T. Entropy production and non-Markovian dynamical maps. Sci. Rep. **7**, 12447 (2017).

S15. Gemmer, J., Michel, M., & Mahler, G. *Quantum Thermodynamics, Lect. Notes Phys. 784* (Springer, Berlin Heidelberg 2009).

S16. Kieu, T. D. The Second Law, Maxwell's Demon, and Work Derivable from Quantum Heat Engines. Phys. Rev. Lett. **93**, 140403 (2004).

S17. Thomas, G. & Johal, R. S. Quantum Otto engine with exchange coupling in the presence of level degeneracy. Phys. Rev. E **83**, 031135 (2011).

S18. Thomas, G., Siddharth, N., Banerjee, S., & S. Ghosh, Thermodynamics of non-Markovian reservoirs and heat engines. Phys. Rev. E **97**, 062108 (2018).

S19. Xiong, H. N., Zhang, W. M., Wang, X. G., & Wu, M. H. Exact non-Markovian cavity dynamics strongly coupled to a reservoir. Phys. Rev. A **82** 012105 90 (2010).

S20. Zhang, X. Y., Huang, X. L., & Yi, X. X. Quantum Otto heat engine with a non-Markovian reservoir. J. Phys. A: Math. Theor. **47**, 455002 (2014).

S21. Paavola, J., Piilo, J., Suominen, K.-A., & Maniscalco, S. Environment-dependent dissipation in quantum Brownian motion. Phys. Rev. A **79**, 052120 (2009).

S22. Intravaia, F., Maniscalco, S., & Messina, A. Density-matrix operatorial solution of the non-Markovian master equation for quantum Brownian motion. Phys. Rev. A **67**, 042108 (2003).

S23. Goan, H. S., Chen, P. W., & Jian, C. Non-Markovian finite-temperature two-time correlation functions of system operators. J. Chem. Phys. **134**, 124112 (2011).

S24. Landauer, R. Information is physical. Physics Today, 44(5), 23-29 (1991).

S25. Koski, J.V., Maisia, V. F., Pekolaa, J. P., & Averind, D. V. Experimental realization of a Szilard engine with a single electron. PNAS **111**, 13786 (2014).

S26. Deffner, S., & Jarzynski, C. Information Processing and the Second Law of Thermodynamics: An Inclusive, Hamiltonian Approach. Phys. Rev. X **3**, 041003 (2013).

S27. Karnani, M., Pääkkönen, K., & Annila, A. Decoherence and the Transition from Quantum to Classical. Pro. R. Soc. A **465** (2107), 2155-2175 (2009).

S28. Vedral, V. *Decoding reality: The universe as quantum information* (Oxford University Press (2010)).

S29. Vedral, V. Information and physics. Information, 3(2), 219-223 (2012).

S30. Davies, P., & Gregersen, N. H. *Information and the nature of reality: from physics to metaphysics* (Cambridge University Press (2010)).

S31. del Rio, L., Åberg, J., Renner, R., Dahlsten, O. and Vedral, The thermodynamic meaning of negative entropy. Nature **474**, 61-63 (2011).

S32. Sagawa, T. & Ueda, M. Second Law of Thermodynamics with Discrete Quantum Feedback Control. Phys. Rev. Lett. **100**, 080403 (2008).

S33. Landauer, R. Irreversibility and heat generation in the computing process. IBM J. Res. Dev. **5**, 183 (1961).

S34. De Donder, T., Van Rysselberghe, P., *Affinity* (Stanford University Press: Menlo Park, CA. 1936)

S35. Nielsen, M. A., Caves, C. M., Schumacher, B., & Barnum, H. Information-theoretic approach to quantum error correction and reversible measurement. Proc. R. Soc. A **454**, 277 (1998).

S36. Parrondo, J. M. R., Horowitz, J. M., & Sagawa, T. Thermodynamics of information. Nature Phys. **11**, 131-139 (2015).

S37. Lloyd, S. Use of mutual information to decrease entropy: Implications for the second law of thermodynamics. Phys. Rev. A **39**, 5378-5386 (1989).

S38. Sagawa, T. Second law, entropy production, and reversibility in thermodynamics of information. arXiv:1712.06858.

S39. Jung Jun Park, Kang-Hwan Kim, Sagawa, T., & Sang Wook Kim, Heat Engine Driven by Purely Quantum Information. Phys. Rev. Lett. **111**, 230402 (2013).

S40. Li-Hang Ren & Heng Fan, Second law of thermodynamics with quantum memory. Phys. Rev. A **96**, 042304 (2017).

S41. Jarzynski, C. Nonequilibrium Equality for Free Energy Differences. Phys. Rev. Lett. **78**, 2690 (1997).

S42. Crooks, G. E. Entropy production fluctuation theorem and the nonequilibrium work relation for free energy differences. Phys. Rev. E **60**, 2721 (1999).

S43. Parrondo, J. M. R., Van den Broeck, C., & Kawai, R. Entropy production and the arrow of time. New J. Phys. **11**, 073008 (2009).

S44. Deffner, S., & Lutz, E. Generalized Clausius Inequality for Nonequilibrium Quantum Processes. Phys. Rev. Lett. **105**, 170402 (2010).

S45. Esposito, M., & Van den Broeck, C. Second law and Landauer principle far from equilibrium. EPL, **95** (2011) 40004.

S46. Plastina, F., Alecce, A., Apollaro, T. J. G., Falcone, G., Francica, G., Galve, F., Lo Gullo, N., & Zambrini, R. Irreversible Work and Inner Friction in Quantum Thermodynamic Processes. Phys. Rev. Lett. **113**, 260601 (2014).

S47. Batalhão, T. B., Souza, A. M., Sarthour, R. S., Oliveira, I. S., Paternostro, M., Lutz, E., & Serra, R. M. Irreversibility and the Arrow of Time in a Quenched Quantum System. Phys. Rev. Lett. **115**, 190601 (2015).

S48. Francica, G., Goold, J., & Plastina, F. Role of coherence in the nonequilibrium thermodynamics of quantum systems Phys. Rev. E **99**, 042105 (2019).

S49. Deffner, S., & Campbell, S. Quantum speed limits: from Heisenberg's uncertainty principle to optimal quantum control. J. Phys. A: Math. Theor. 50, 453001 (2017).

S50. Batalháo, T. B., Gherardini, S., Santos, J. P., Landi, G. T., & Paternostro, M. *Characterizing Irreversibility in Open Quantum Systems* (Springer 2019).

S51. Deffner, S., & Lutz, E. Nonequilibrium Entropy Production for Open Quantum Systems. Phys. Rev. Lett. **107**, 140404 (2011).

S52. Breuer, H. P. & Petruccione, F. *The theory of open quantum systems* (Oxford University Press, Oxford, 2002).

S53. Alicki, R. The quantum open system as a model of the heat engine. J. Phys. A **12**, L103 (1979).

S54. Rivas, A., Huelga, S. F., & Plenio, M. B. Quantum non-Markovianity: characterization, quantification and detection. Rep. Prog. Phys. **77**, 094001 (2014).
